# A novel 8-bp duplication in *ADAT3* causes mild intellectual disability

**DOI:** 10.1038/s41439-018-0007-9

**Published:** 2018-05-21

**Authors:** Ahmad Reza Salehi Chaleshtori, Noriko Miyake, Mohammad Ahmadvand, Oranous Bashti, Naomichi Matsumoto, Mehrdad Noruzinia

**Affiliations:** 1 Department of Medical Genetics, Faculty of Medical Sciences, Tarbiat Modares University, P.O. Box 14115-331 Tehran, Iran; 2 Department of Human Genetics, Yokohama City University Graduate School of Medicine, Yokohama, 236-0004 Japan; 3 Hematology, Oncology and Stem Cell Transplantation Research Center, Shariati Hospital, Tehran University of Medical Sciences, Tehran, P.O. Box 14114 Iran; 4 Laboratory of Medical Genetics, iHealth Clinics, P.O. Box 1913874416 Tehran, Iran

## Abstract

Inosine is a base located at wobble position 34 of the tRNA anticodon stem–loop, enabling the recognition of more than one codon in the translation process. A heterodimer consists of ADAT3 and ADAT2 and is involved in the adenosine-to-inosine conversion in tRNA. Here, we report the second novel *ADAT3* mutation in a patient with microcephaly, intellectual disability, and hyperactivity. These findings constitute a second mutation and expand the clinical spectrum of extremely rare *ADAT3* mutations.

Adenosine (A)-to-inosine (I) RNA editing is a post-transcriptional RNA process capable of generating RNA and protein diversity^1^. Inosine at wobble position 34 of tRNA anticodons can translate codons ending in uracil, cytosine, or adenine^2^. The modification, which creates an I from an A at position 34 (wobble position) of tRNA, is catalyzed by the heterodimeric enzyme, adenosine deaminase, tRNA-specific 3 (ADAT3)/ADAT2^2^.

Alazami et al. described a homozygous *ADAT3* mutation (c.382 G > A, p.Val128Met) in 24 affected individuals with autosomal-recessive mental retardation 36 (MRT36; MIM*615286) from eight consanguineous Arab families^3^. Very recently, El-Hattab et al. reported an additional 15 patients with an identical homozygous *ADAT3* mutation in 15 affected individuals from 11 Arab families. In the previous reports, strabismus, microcephaly, failure to thrive, and abnormal brain structure were frequently seen in such patients.

We encountered a 6-year-old female presenting with intellectual disability, mild cognitive impairment, attention deficit, hyperactivity disorder, neurodevelopmental delay, speech delay, and microcephaly. The patient’s face was asymmetric, and her nasal bridge was depressed. She was born to healthy Iranian consanguineous parents (Fig. 1a). The proband visited our genetic center seeking a genetic testing service. Considering the clinical findings, the targeted sequencing of 12 genes associated with microcephaly (*SLC25A19, STIL, ASPM, CEP135, MCPH1, CDK5RAP2, CENPJ, CEP152, WDR62, ZNF335, ADAT3*, and *EFTUD2*) was provided. After obtaining informed consent, genomic DNA of peripheral blood leukocytes was extracted and used for the genome partitioning. Targeted capture was performed using the GeneRead DNAseq Custom Panel V2 (QIAGEN, Hilden, Germany), and the libraries were sequenced to mean >80–100 × coverage on a HiSeq2000 sequencing platform (Illumina, San Diego, CA, USA). For read mapping and variant analysis, sample sequences were aligned to the human reference genome (GRCh37/hg19) using Burrows-Wheeler Aligner^4^. To identify variants relevant to the disease, the obtained data were manipulated using picard and processed with the Genome Analysis Toolkit (GATK refv1.2905)^5^.

**Fig. 1:**
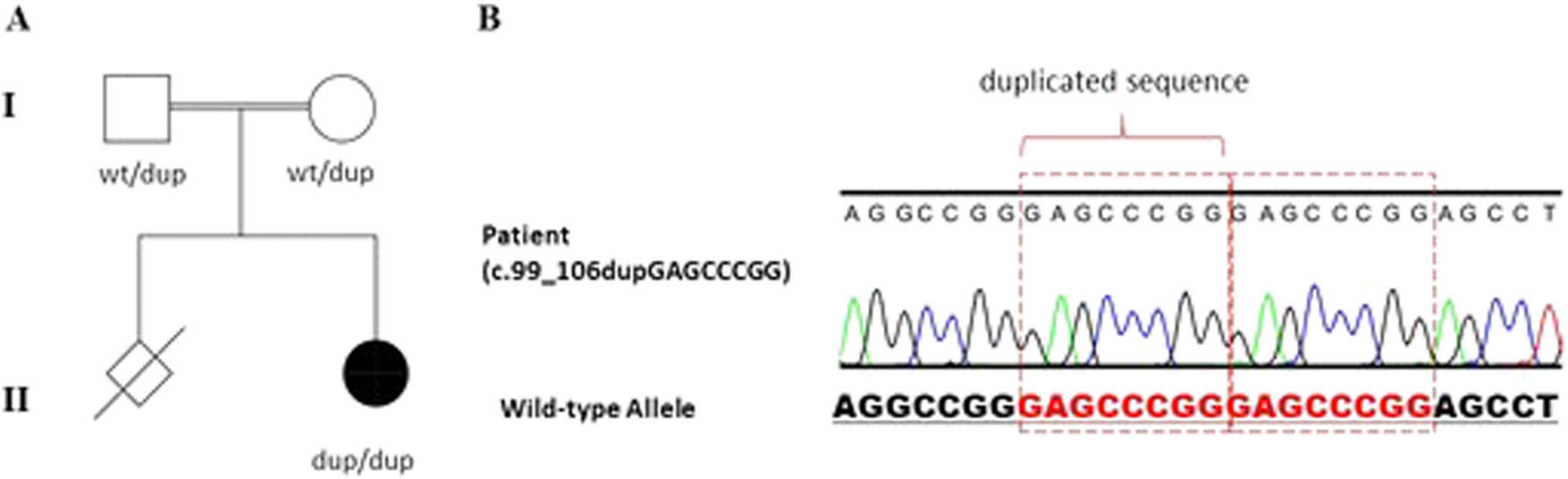
Segregation status of the mutation and Sanger confirmation of c.99_106dupGAGCCCGG mutation in the proband **a** Pedigree information and segregation status of the *ADAT3* 8-bp duplication. **b** Chromatogram of the c.99_106dupGAGCCCGG, p.(Glu36Glyfs*44) mutation

Through our targeted sequencing, we identified a homozygous 8-bp duplication in *ADAT3* (c.99_106dupGAGCCCGG, p.(Glu36Glyfs*44); Fig. 1b). This variant was not previously registered in the 1000 genomes database (http://browser.1000genomes.org/index.html), ExAC browser (http://exac.broadinstitute.org/), or EVS (http://evs.gs.washington.edu/EVS/). Since this gene has one coding exon, the frameshift mutation might produce a truncated protein. We confirmed both parents as heterozygous carriers (Fig. 1b), agreeing with the autosomal-recessive mode of inheritance.

The proband we present here shared many clinical features with the patients reported by El-Hattab and Alazami^3^, including hyperactivity, developmental delay, microcephaly, depressed nasal bridge, and asymmetric face, which were commonly seen in the current patient. In contrast, our patient showed speech delay, while El-Hattab reported speech incapability (no words) in patients with the c.382 G > A mutation. Most patients with the c.382 G > A mutation in *ADAT3* have been characterized with moderate to severe cognitive impairment^3,6^, while the present patient was a sufferer from mild intellectual disability. Moreover, previous reports on *ADAT3* mutation noted strabismus as an accompanying sign of cognitive impairment in patients with the c.382 G > A mutation^3,6^; however, this patient did not show strabismus. Other clinical findings were consistent with previous reports^3,6^ (Table 1); therefore, the difference in clinical features might be due to the different mutational effects of respective mutations.

**Table 1:** Clinical features of the patient compared to previous report of *ADAT3*-related cognitive impairment

This report	Previous report
Cognition	
Intellectual disability	Intellectual disability
Mild to moderate cognitive impairment	Moderate to severe cognitive impairment
Attention deficit hyperactivity disorder (ADHD)	Aggressive/hyperactivity
Development	
Neurodevelopmental delay	Developmental delay
Speech delay	No speech ability
Face–skull	
Microcephaly	Microcephaly
Asymmetric face	Elongated face with prominent nose
Depressed nasal bridge	Depressed nasal bridge
No strabismus	Strabismus

In conclusion, we report a novel and second *ADAT3* mutation in a patient with intellectual disability and propose that *ADAT3* sequencing should be considered for intellectual disability in the Middle East.

## Data Availability

The relevant data from this Data Report are hosted at the Human Genome Variation Database at 10.6084/m9.figshare.hgv.1942.
